# Accumulative Competitive Season Training Stress Affects Neuromuscular Function and Increases Injury Risk in Uninjured D1 Female Athletes

**DOI:** 10.3389/fspor.2020.610475

**Published:** 2021-02-10

**Authors:** Troy M. Purdom, Kyle S. Levers, Jacob Giles, Lindsey Brown, Chase S. McPherson, Jordan Howard

**Affiliations:** ^1^Department of Kinesiology, North Carolina Agriculture and Technical State University, Greensboro, NC, United States; ^2^Department of Exercise and Nutrition Sciences, George Washington University, Washington, DC, United States; ^3^Department of Health, Athletic Training, Recreation, and Kinesiology, Longwood University, Farmville, VA, United States; ^4^Department of Occupational Therapy, Virginia Common Wealth University, Richmond, VA, United States

**Keywords:** Y-balance, tonicity, mobility, stability, muscle tone, injury, peripheral nervous system, central nervous system

## Abstract

Previous research has shown that acute competition training stress negatively affects neuromuscular function which can perpetuate a predisposition to injury. This study's aim was to investigate the effect of accumulated competition training stress effect on neuromuscular function and incidence of increased injury risk in uninjured female D1 soccer players. Neuromuscular function was evaluated in fifteen female division I soccer athletes who played >85% of competitive season competitions who were tested for mobility/stability, leg length symmetry, and vertical power at three different points across the competitive season (pre, mid, and post time blocks). Leg length symmetry was measured from the anterior superior iliac spine to the lateral malleolus prior to Y-balance testing. The Y-balance testing measures unilateral anterior, posteromedial, and posterolateral reach achieved in single leg stance using metrics that include L/R normalized composite reach (NCOMP), L/R normalized antiorior reach (NANT), and L/R NCOMP/NANT segmental differences across time. Injury risk was evaluated using validated objective criteria that included: (NCOMP total reach <94% of limb length^*^3), (NANT reach distance <84% leg length) along with NCOMP and NANT asymmetries >4.0. Maximal vertical power (MVP) was measured via vertical jump. Multiple repeated measures ANOVAs evaluated NCOMP, NANT, MVP, and leg length symmetry across time with LSD *post hoc* testing when relevant (X ± SD). A significant main effect was found [*F*_(1, 14)_ = 62.92, *p* < 0.001; η^2^ =0.82] with training stress and neuromuscular function without affecting maximal vertical power. Eighty percent of subject's bilateral NCOMP scores fell below the YBT reach standard at midseason (ES = 0.95, *p* = 0.02) while all subjects NANT reach distance remained below the reach threshold (ES = 0.74, *p* = 0.003) indicating a 6.5× and 2.5× greater injury risk, respectively. Competition stress affected neuromuscular function without affecting maximal power, which negatively impacted stability and increased injury risk.

## Introduction

Competitive athletes regularly endure high training loads that consist of repetitive exercise at high intensities, volumes, and frequencies with inadequate rest periods (Bengtsson et al., 2013; Dubois et al., 2018; Walker et al., 2019; Satkunskiene et al., 2020) that can perpetuate chronic fatigue and eventually injury (Silva et al., 2016; Jones et al., 2017). Furthermore, a dose response exists with increased frequency and duration further exacerbating the training stress throughout the competitive season (Bengtsson et al., 2013; Dubois et al., 2018; Caterisano et al., 2019; Satkunskiene et al., 2020). The high intensity, duration, and frequency of physical stress that collegiate athletes endure during training and competition can have residual effects (Bengtsson et al., 2013; Jones et al., 2017; Dubois et al., 2018; Walker et al., 2019; Satkunskiene et al., 2020) disrupting systematic neuromuscular function (Needle et al., 2014; Brownstein et al., 2017; Thomas et al., 2018; Satkunskiene et al., 2020) specifically, both central and peripheral fatigue for up to 72 and 48 h respectively (Brownstein et al., 2017; Thomas et al., 2018). Furthermore, the residual fatigue that competitive soccer athletes experience manifests as reduced performance prowess and increased injury rate when adequate recovery is not provided (Bengtsson et al., 2013; Silva et al., 2016; Dubois et al., 2018; Caterisano et al., 2019). In an 11 year retrospective study, competitive soccer matches where the team lost were significantly higher with <3 days of recovery while athletes with <4 days of recovery were shown to have increased rates of soft tissue injury (Bengtsson et al., 2013). Moreover, neuromuscular dysfunction due to cellular and tissue damage incurred during competition (Jones et al., 2017; Walker et al., 2019) has been shown to contribute to diminished neuromechanical function (Nédélec et al., 2012; Bengtsson et al., 2013; Brownstein et al., 2017; Satkunskiene et al., 2020) or stability (Plisky et al., 2006) that can manifest as a lower extremity injury (Plisky et al., 2006; Needle et al., 2014; Brazier et al., 2019; Higashihara et al., 2019).

Stability is contingent on motor control as a function of the afferent and efferent somatic nervous system function. Moreover, postural control relies on the integration from multiple control centers which include proprioception, visual cues, vestibular input, and planning (Needle et al., 2014). While sensory feedback is necessary for stability, motor control is largely a function of systematic neuromuscular function (Needle et al., 2014). Furthermore, postural control is highly contingent on the agonist/antagonist co-contraction to increase joint stiffness (Needle et al., 2014; Brazier et al., 2019) and is maintained through muscle tone, or low-level steady-state muscular contraction (Needle et al., 2014). Chronically stressing the nervous system can have negative impacts on neuromuscular function (Bengtsson et al., 2013; Buckthorpe et al., 2014; Brownstein et al., 2017; Thomas et al., 2018; Higashihara et al., 2019), affecting muscle tone and limiting mobility/stability due to hypertonic muscle spindles (afferent sensory network) that stimulate the reflex arc (Masi and Hannon, 2008; Needle et al., 2014; Stecco et al., 2014) and therefore muscle contraction (Bengtsson et al., 2013). For instance, while postural control is maintained by muscle tone, hypertonicity, or an abnormal increase in muscle tone with resistance to active movement can negatively affect joint stability (Needle et al., 2014). Furthermore, excessive hypertonicity can increase antagonist (opposing) muscle tone (Bengtsson et al., 2013; Needle et al., 2014; Stecco et al., 2014) which can limit joint range of motion and therefore mobility. Interestingly, increased joint stiffness may improve stability (Brazier et al., 2019) while failure to regulate stiffness can contribute to injury (Needle et al., 2014; Satkunskiene et al., 2020) through unfavorable motor control at specific joint centers.

Passive muscle tone is a neuromuscular function that provides low level resistance to stretch in an effort to maintain postural stability (equitable agonist/antagonist tension) (Masi and Hannon, 2008; Stecco et al., 2014; Higashihara et al., 2019). The degradation of motor control [balance] (Plisky et al., 2006) can occur acutely due to peripheral fatigue as a result of training stress (Brownstein et al., 2017; Satkunskiene et al., 2020) which can negatively affect postural stability. However, chronic training can influence the tissues surrounding the muscle (myofascial and connective tissue networks) which can negatively affect skeletal muscle function (Masi and Hannon, 2008; Nédélec et al., 2012; Needle et al., 2014; Stecco et al., 2014; Satkunskiene et al., 2020). Moreover, a peripheral complication of increased passive muscle tone is fascial rigidity which can hyper-stimulate the muscle spindle (Stecco et al., 2014), also known as hypertonicity (Needle et al., 2014). Fascial rigidity can create a positive feedback loop with the dysfunction of the muscle spindle (Plisky et al., 2006; Nédélec et al., 2012) hyperactivating passive muscle tone which reduces compliance of the antagonist muscle (Stecco et al., 2014). Therefore, reduced muscle compliance due to increased passive muscle tone can result in poor stability, and thus limit mobility (Plisky et al., 2006; Needle et al., 2014; Gonell et al., 2015). The negative effects of reduced muscle compliance can manifest as a reduction in stride length (Higashihara et al., 2019; Satkunskiene et al., 2020), postural degradation during high velocity movements (Masi and Hannon, 2008; Iwamoto et al., 2017) amongst other negative outcomes significantly affecting neuromechanical function during sport.

The high training loads collegiate soccer players experience across the competitive season without adequate recovery have demonstrated deleterious effects on neuromuscular function (Bengtsson et al., 2013; Brownstein et al., 2017; Satkunskiene et al., 2020) and therefore warrant periodic neuromuscular function evaluation (Gonell et al., 2015; Stiffler et al., 2017). A recent study detailing limitations of pre-season mobility/stability function to determine non-contact injuries across the season further suggests that periodic evaluation of neuromuscular function is necessary (Luedke et al., 2020). Specifically, validated measures of neuromuscular function can be used to monitor applied mobility-stability functionality (Plisky et al., 2006; Gonell et al., 2015; Stiffler et al., 2017) and maximal force/power production (Nédélec et al., 2012; Buckthorpe et al., 2014; Brownstein et al., 2017; Thomas et al., 2018). The Y-balance test (YBT) has been shown to be a valid method to evaluate stability asymmetries and therefore neuromuscular function in athletic populations (Plisky et al., 2006; Gonell et al., 2015; Giles et al., 2017; Stiffler et al., 2017), while maximal vertical power (MVP) is a valid method of measuring lower extremity maximal force production (Canavan and Vescovi, 2004; Quagliarella et al., 2011; Buckthorpe et al., 2014; Thomas et al., 2018). Lower body asymmetry is defined as a discrepancy between the right and left (L/R) limb reach distance measured actively where passive measurement includes limb leg length, or the distance from the anterior superior iliac crest and lateral malleolus (Plisky et al., 2006). Anterior reach considers ankle dorsiflexion while posteromedial reach is a useful tool to identify ankle perturbation and therefore instability, specifically the anterior tibialis as well as lower extremity coordination (Plisky et al., 2006; Stiffler et al., 2017). The decrement in active single stance reach and therefore range of motion evaluated by the YBT can indicate a greater risk of injury (Plisky et al., 2006; Gonell et al., 2015; Stiffler et al., 2017) due to neuromuscular dysfunction (Masi and Hannon, 2008; Needle et al., 2014; Brownstein et al., 2017; Higashihara et al., 2019; Satkunskiene et al., 2020). Therefore, the aim of this study was to evaluate the effect of accumulative competitive season stress on neuromuscular function, specifically: measures of mobility/stability, asymmetry, and MVP in uninjured, high-minute Division I athletes.

## Materials and Methods

### Exercise Design

**Figure 1:**
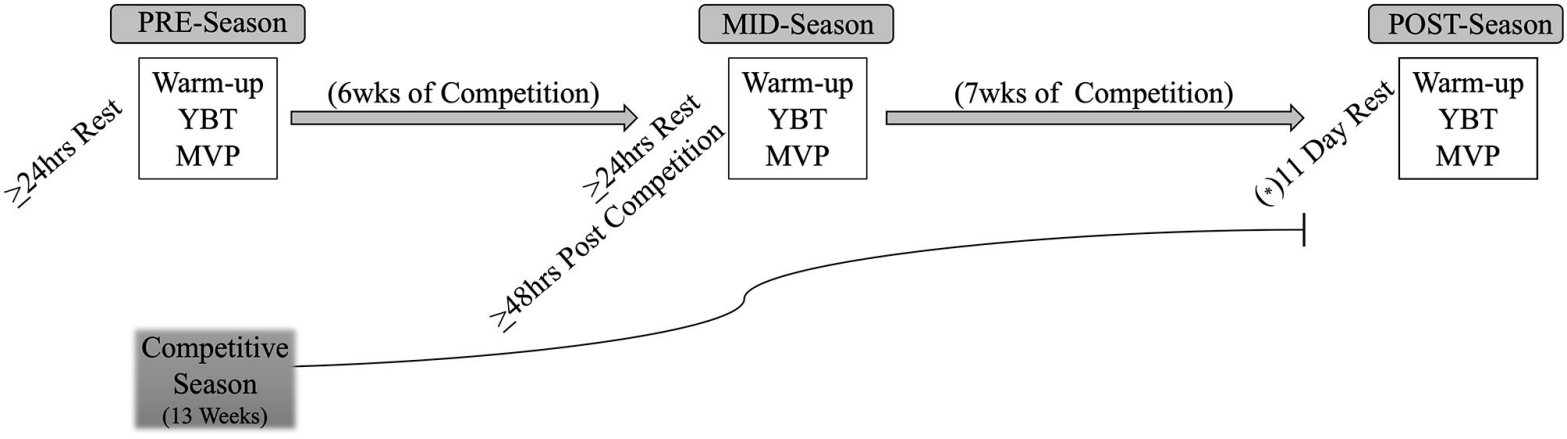
Study timeline including data collection time blocks. (*) NCAA mandated post season discretionary period (NCAA D1 2018-2019 Manual) (The National Collegiate Athletic Association, 2018); POST season data was collected 11 days after the last competitive match. (YBT) y-balance Testing; (MVP) Maximal Vertical Power; (>)greater than or equal to.

Using a repeated measures design, this study investigated the impact of accumulated collegiate competitive season training stress on neuromuscular function in Division I female soccer players. Sample size was determined using a repeated measures a priori power analysis (G^*^Power, version 3.1.9.6) indicating a sample size of (*n* = 9) based upon η^2^= 0.391 from Satkunskiene et al. 2020 (Satkunskiene et al., 2020). Testing occurred at pre-designated testing blocks across the competitive season: pre-season (PRE), mid-season (MID) and post-season (POST) as shown in Figure 1. The PRE:MID season time points occurred six weeks apart while the MID:POST season time points were nine weeks apart. Division I NCAA compliance regulations have a seven day minimum discretionary rule preventing student athlete participation in, “all athletic related activities… beginning the day after… the last contest of the championship segment” (The National Collegiate Athletic Association, 2018). Therefore, POST season testing occurred 11 days after the final competitive match extending the POST testing block. No prescribed exercise occurred during the 11-day period between the last competition and POST time block and all testing was conducted with >24 h of rest and >48 h post competition.

### Subjects

Twenty-nine Division I female soccer athletes initially enrolled in the study providing informed written consent approved by the university institutional review board and completed a health history questionnaire prior to participation. This study was conducted in accordance with the Declaration of Helsinki. Accumulation of competitive match stress paired with lack of adequate recovery have been shown to incur significant physiological stress that can accumulate (Bengtsson et al., 2013; Jones et al., 2017; Dubois et al., 2018; Walker et al., 2019; Satkunskiene et al., 2020) with deleterious effects that result in injury (Bengtsson et al., 2013; Needle et al., 2014; Caterisano et al., 2019). Therefore, the inclusion criteria consisted of high minute players with >5yrs competitive experience who incurred significant competition training stress by playing in >85% of competitive season games (>18/21) throughout the 13-week collegiate season, and had no significant injury preventing testing compliance. During the 13wk competitive season, subjects participated in concurrent training (mixed resistance and endurance training) sessions 4-5 days per week with a minimum of two matches per week, which has been shown to incur significant stress that can accumulate across the competitive season (Bengtsson et al., 2013; Jones et al., 2017; Dubois et al., 2018; Walker et al., 2019; Satkunskiene et al., 2020). Total weekly training volume throughout the competitive season could not exceed 20 h per NCAA Guidelines (The National Collegiate Athletic Association, 2018). However, the conference game schedule included ~3 days recovery between matches, which has been shown to promote injury (Bengtsson et al., 2013). Of the 29 subjects who initially enrolled, three subjects voluntarily dropped from the study, two were excluded due to injury preventing testing, and nine excluded due to playing <85% of competitions throughout the competitive season. Thus, demographic information for 15 high minute uninjured Division I female soccer players included are shown in Table 1.

**Table 1:** Demographic information for subjects (*n* = 15) across all three time blocks (PRE, MID, POST).

	**Age**	**Height**	**Weight**	**%BF**	**FFM**	**FM**	**Played/Total**
	**(years)**	**(cm)**	**(kg)**	**(%)**	**(kg)**	**(kg)**	**(Min)**
PRE	19.5 ± 1.1	—	60.0 ± 5.1	18.7 ± 3.8	48.7 ± 3.7	11.3 ± 2.9	—
MID	19.6 ± 1.2	165.6 ± 4.3	60.7 ± 4.6	18.3 ± 3.2	49.5 ± 3.1	11.2 ± 2.6	481 ± 194/850
POST	19.6 ± 1.2	166.1 ± 5.2	60.3 ± 5.2	19.0 ± 3.0	48.8 ± 4.0	11.5 ± 2.3	1,251 ± 495/2,010

*Data are presented as mean ± SD. PRE, pre-season; MID, midseason; POST, post-season. (Played/Total) ratio of competition minutes played to total competition minutes across 21 competitive season games. BF, body fat; FFM, fat free mass; FM, fat mass*.

**Figure 2:**
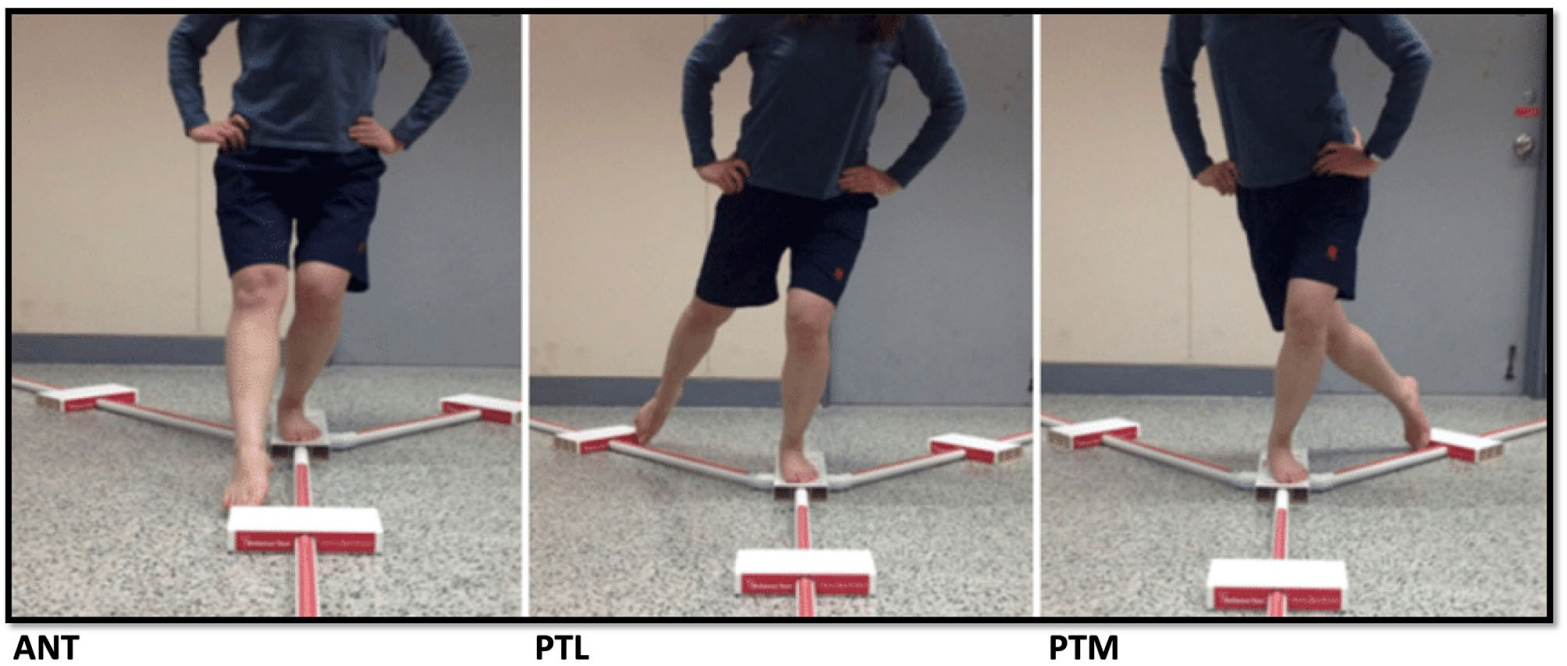
Illustration of the single leg Y-balance test (YBT) in the anterior (ANT), posterior lateral (PTL), and posterior medial (PTM) directions. Injury risk indicators were NCOMP reach deficiency (total reach <94% of limb length*3), NANT reach deficiency (ANT reach distance <84% leg length), and segmental comparisons >4.0 (Plisky et al., 2006; Gonell et al., 2015; Stiffler et al., 2017).

Each testing block began with subjects arriving to the laboratory having refrained from exercise for >24 h, avoiding stimulants/depressants including caffeine for >12 h, and fasted for >4 h. Height and weight were measured via electronic standiometer and scale (Seca Corp. Chino, CA, USA) while tissue density was measured by a trained researcher using handheld skinfold calipers (Beta Technology, Santa Cruz, CA, USA) and a three site skin fold method along with the Brozek equation to estimate body composition (American College of Sports Medicine, 2018). The subjects then completed a 5-min cycling aerobic warm-up prior to completing a standardized dynamic warm-up (20 m of high knees, butt kickers, and high bounds). Neuromuscular function was assessed with mobility/stability and joint symmetry utilizing a metric measuring tape and a YBT testing apparatus (Functional Movement Systems, Chatham, VA) by multiple trained researchers, which has been shown to have a high degree of interrater reliability (Shaffer et al., 2013). Joint symmetry was first evaluated by measuring leg lengths from the anterior superior iliac spine to the lateral malleolus followed by recording the best of three YBT attempts per side without shoes (Plisky et al., 2006; Gonell et al., 2015). The YBT measures anterior, posterolateral, and posteromedial reach distance by requiring the subject to maintain single leg stance while pushing the reach indicator with their non-stance foot in each of the three aforementioned directions (Figure 2). The stance foot and non-stance foot must remain in contact with the YBT apparatus at all times. The stance foot heel must not raise from the YBT apparatus while the toes of the non-stance foot maintain contact with the reach indicator until the subject can no longer progress the reach indicator or maintain balance. The subject must then return to the start position under control without touching the ground. Measures of the YBT are further discussed and shown below which include leg length symmetry (LLSYM) (difference between right and left leg lengths), the left-right (L/R) normalized composite reach (NCOMP), L/R normalized anterior reach (NANT), and L/R NCOMP/NANT segmental comparisons (Plisky et al., 2006; Gonell et al., 2015; Stiffler et al., 2017). Previous validated metrics that specify a predisposition to injury risk were identified as NCOMP reach deficiency (<94% leg length^*^3), NANT reach deficiency (<84% leg length) (Plisky et al., 2006; Stiffler et al., 2017), and/or L/R NCOMP/NANT (side-to-side comparison >4.0) (Plisky et al., 2006; Gonell et al., 2015; Stiffler et al., 2017). Therefore, NCOMP (%) and NANT (%) reach threshold values along with L/R side-to-side difference comparisons (absolute value) were used as injury risk indicators across time blocks.

YBT Measures:

- LLSYM = side-to-side comparison of anterior, superior iliac spine to lateral malleolus measured in millimeters (mm)
- NCOMP = [(ANT+PTL+PTM/leg length^*^3)^*^100] measured as a (%)
- NANT = [(ANT/ leg length)^*^100] measured as a (%)
- L/R NCOMP segmental comparisons (absolute value)
- L/R NANT segmental comparisons (absolute value)

Injury Risk Indicators:

- NCOMP reach deficiency (total reach <94% of limb length^*^3)
- NANT reach deficiency (anterior reach distance <84% leg length)
- L/R NANT side-to-side comparison >4.0 (absolute value)
- L/R NCOMP side-to-side comparison >4.0 (absolute value)

LLSYM: leg length symmetry; NCOMP: normalized composite score; ANT: anterior reach distance; PTL, post-eriorlateral reach distance; PTM, post-eriormedial reach distance; NANT, normalized anterior reach score.

After the warm-up protocol and YBT, MVP function was evaluated using vertical jump performance (Thomas et al., 2018) with a Vertec apparatus (Sports Imports, Columbus, OH) (Canavan and Vescovi, 2004; Quagliarella et al., 2011; Nédélec et al., 2012). Standing reach height was first measured prior to subjects completing three maximal effort countermovement jumps with a 60 s rest between attempts. Of the three maximal vertical jumps, the highest jump was recorded (Canavan and Vescovi, 2004). Vertical jump heights were converted to watts and therefore MVP using the Harman Formula: Power (watts) = [61.9^*^jump height (cm)] + [36.0^*^body mass (kg)] – 1,822] (Canavan and Vescovi, 2004; Quagliarella et al., 2011).

### Statistical Analysis

Neuromuscular function was evaluated using MVP (watts) (Canavan and Vescovi, 2004; Quagliarella et al., 2011; Thomas et al., 2018) along with the YBT (NCOMP, NANT, LLSYM) (Plisky et al., 2006; Gonell et al., 2015). Two repeated measures analysis of variance (ANOVA) 4 × 3 statistical models (dependent variable × time) were used to analyze dependent variables. Metrics from the YBT and MVP were used comparatively to assess neuromuscular functional changes across the competitive season (PRE, MID, and POST time blocks): NCOMP L/R comparison difference (absolute value), NANT L/R comparison difference (absolute value), LLSYM, and MVP. The second ANOVA model compared left and right limb NCOMP reach distance as well as L/R limb NANT reach distance; both expressed as a percentage of leg length with the equations specified above across the competitive season using SPSS version 23 (IBM Corp, Armonk, NY). When statistical relevance was observed, the LSD *post hoc* test was used to evaluate pairwise comparisons. Significance was set at (*p* < 0.05) and all values are represented as (mean ± SD) and effect size (ES).

## Results

**Figure 3:**
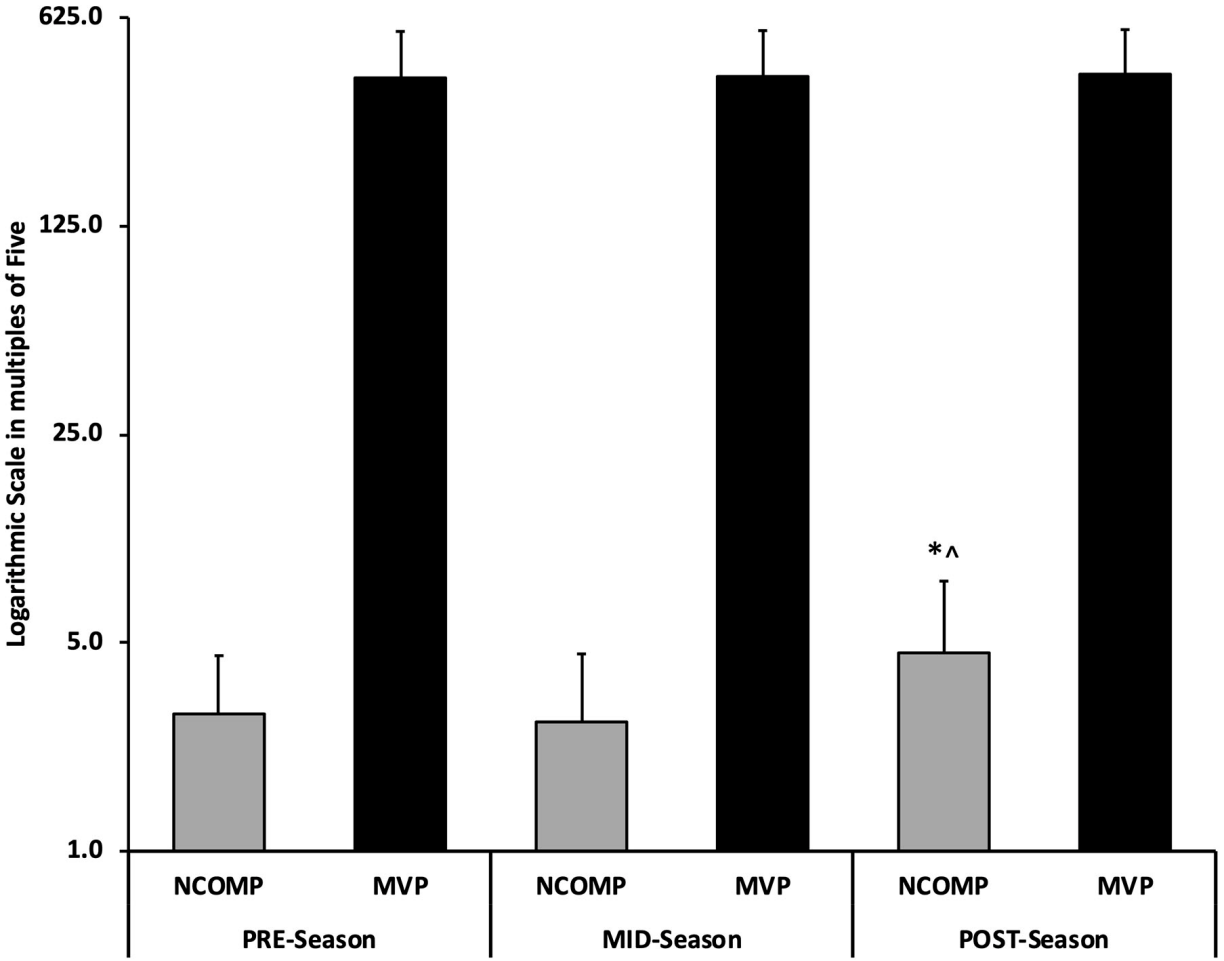
Relationship between maximal vertical power (MVP) expressed in watts (W) and NCOMP left/right difference expressed as an (absolute value) across the competitive season (PRE, MID, POST time blocks). (NCOMP) normalized composite score, NCOMP = [(ANT+PTL+PTM/(leg length*3)]*100. (*) indicates a statistically significant change from MID season (*p* < 0.05). (∧) left/right NCOMP difference exceeds 4.0 indicating a 6.5x increase in injury risk (Plisky et al., 2006; Gonell et al., 2015; Stiffler et al., 2017).

**Figure 4:**
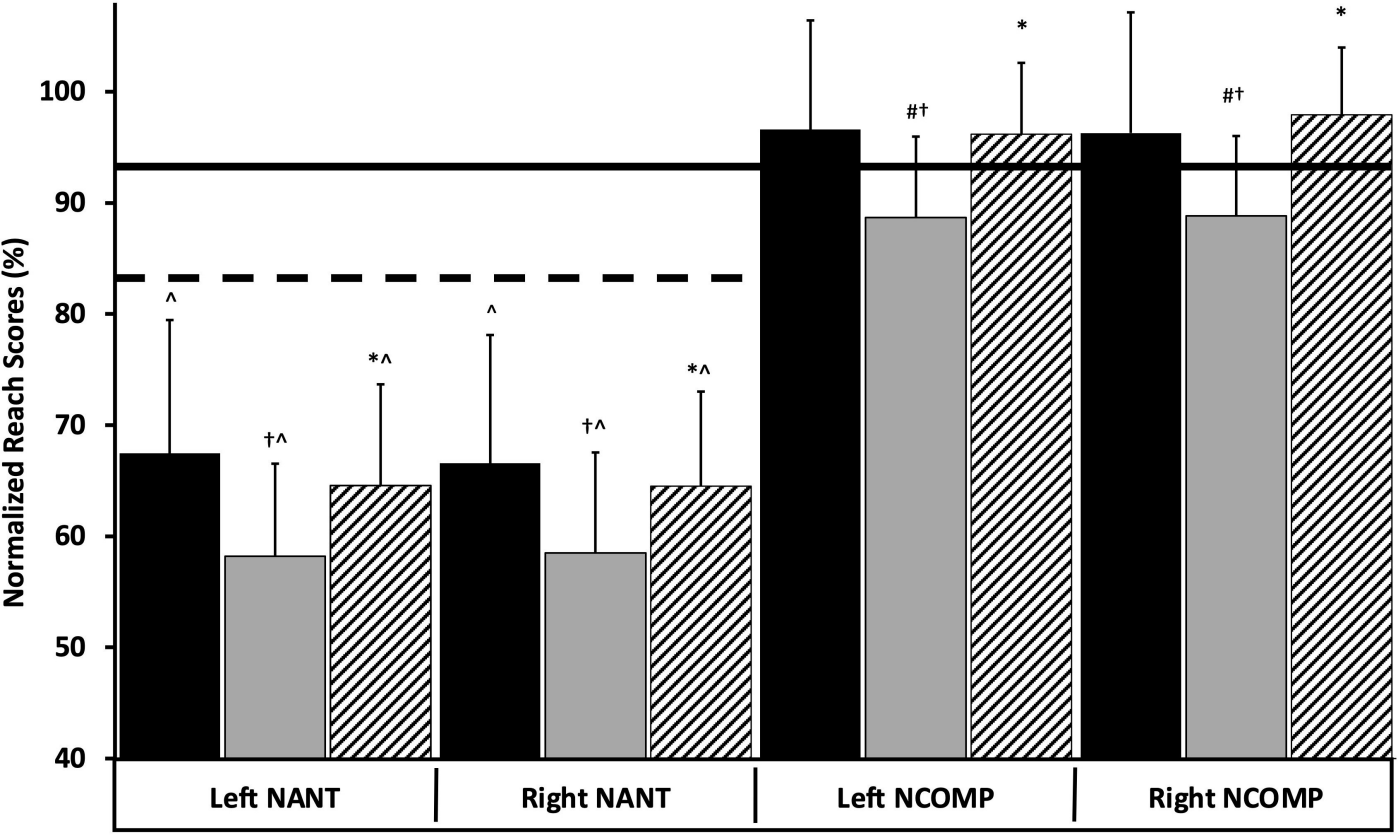
Left and right normalized anterior reach (NANT) and normalized composite scores (NCOMP) scores expressed as percentages and displayed across the competitive season [PRE-season (black), MID-season (gray) POST-season (striped)]. (†) indicates a statistically significant change from PRE-season (*p* < 0.05). (*) indicates a statistically significant change from MID-season (*p* < 0.05). (#) indicates failure to meet the NCOMP relative injury risk threshold = [(ANT+PTL+PTM) <94% of (limb length*3)] which is visually represented by the solid horizontal line (Plisky et al., 2006). (∧) indicates failure to meet the NANT relative injury risk threshold = (ANT reach distance <84% of limb length), which is visually represented by the dashed horizontal line (Plisky et al., 2006; Gonell et al., 2015; Stiffler et al., 2017). ANT, anterior; PTL, posterior lateral; PTM, posterior medial.

A significant main effect was observed [*F*_(1, 14)_ = 62.29, *p* < 0.001; η^2^ = 0.82] with accumulated competition stress and neuromuscular function across time in both MID and POST timepoints in uninjured D1 female soccer players shown in Table 2. Pairwise comparisons revealed neuromuscular function was affected by accumulated competition training stress across the competitive season through a significant increase in L/R NCOMP segmental difference MID-POST (MID: 2.7 ± 1.9, POST: 4.6 ± 3.4; *p* = 0.05; ES = 0.69) without relevant differences in MVP shown in Figure 3. Left-right NCOMP and NANT reach distances were also impacted by competitive training stress across time [*F*_(1, 14)_ = 2.79, *p* < 0.01; η^2^ = 0.30]. NCOMP and NANT reach distance for both legs decreased from PRE:MID, and then increased from MID:POST shown in Figure 4.

**Table 2:** Dependent variables are shown across all testing blocks (competitive season).

**Dependent variables**	**PRE-season**			**MID-season**			**POST-season**		
	**LEFT**	**RIGHT**	**Abs DIFF**	**LEFT**	**RIGHT**	**Abs DIFF**	**LEFT**	**RIGHT**	**Abs DIFF**
NANT (%)	67.5 ± 12.0∧	66.6 ± 11.5∧	3.7 ± 2.5	58.2 ± 8.3†∧	58.5 ± 9.0†∧	3.3 ± 2.1	64.6 ± 9.1*∧	64.5 ± 8.5*∧	4.6 ± 4.1*§
NCOMP (%)	96.6 ± 9.8	96.3 ± 10.9	2.9 ± 1.6	88.7 ± 7.3†#	88.8 ± 7.2†#	2.7 ± 1.9	96.2 ± 6.4*	97.9 ± 6.1*	4.6 ± 3.4*§
LLSYM L/R Diff (cm)	0.9 ± 0.2			1.0 ± 0.3			1.0 ± 0.2		
MVP (W)	392.2 ± 43.7			396.6 ± 43.5			403.6 ± 42.8		

Data are presented as Mean ± SD. NCOMP- normalized composite reach score represented as a percent (%). NANT- normalized anterior reach score represented as a percent (%). LLSYM- leg length symmetry in centimeters (cm). MVP- maximal vertical power in watts (W). Abs Diff- Absolute left/right segmental difference (absolute value). (†) indicates a statistically significant change from PRE-season (p < 0.05). (*) indicates a statistically significant change from MID-season (p < 0.05). (∧)indicates failure to meet the NANT relative injury risk threshold = (ANT reach distance <84% of limb length). (#)indicates failure to meet the NCOMP relative injury risk threshold = [(ANT+PTL+PTM) <94% of (limb length*3)] (Plisky et al., 2006). (§) left/right difference exceeds 4.0 indicating increased injury risk (Plisky et al., 2006; Gonell et al., 2015; Stiffler et al., 2017).

Table 2 details NANT and NCOMP comparisons across each time point. YBT measures indicate that neuromuscular function changed longitudinally with a ~13% decrease in L/R NANT (ES = 0.83) PRE:MID before increasing 9.5% MID:POST (ES = 0.71). Longitudinal changes were further detailed with L/R NCOMP reach distance reducing ~8% PRE:MID (ES = 0.86) and then increasing ~8.5% (ES = 0.96). The accumulated training stress reduced bilateral NCOMP reach distance PRE:MID where 80% of subjects (12/15) had NCOMP reach distance scores below the YBT injury risk threshold (reach <94% of 3 × limb length) increasing injury risk by 6.5 × (Figure 4). However, bilateral NCOMP reach distance returned above the risk threshold at POST with extended rest (11-days rest prior to POST-season testing). Furthermore, while 100% of the population was at a 2.5 × increased injury risk (NANT reach <84% limb length) for both limbs throughout all three test blocks (entire competitive season) (Figure 4), bilateral NANT reach distance paralleled the NCOMP significant decrease PRE-MID that increased with significant rest MID:POST. No significant differences between MVP were observed throughout the competitive season indicating that maximal power production was unaffected by competitive season training stress (*p* = 0.75) (Table 2; Figure 3) with >24 h of rest and >48 h post competition.

## Discussion

The purpose of this study was to evaluate the accumulation of stress of the competitive season on neuromuscular function in Division I female soccer players. To our knowledge, this is the first study to evaluate the effects of accumulated competitive season training stress effect on injury risk as it relates to applied neuromuscular function in uninjured female athletes. Previous research evaluated neuromuscular function acutely (Plisky et al., 2006; Buckthorpe et al., 2014; Gonell et al., 2015; Brownstein et al., 2017; Stiffler et al., 2017; Higashihara et al., 2019) and/or the efficacy of YBT performance and its efficacy to predict injury (Plisky et al., 2006; Shaffer et al., 2013; Gonell et al., 2015; Stiffler et al., 2017). This study highlights the necessity to monitor the neuromuscular system's adaptive capacity to maintain mobility/stability despite continual high intensity, high volume competitive stress. The study sample, which was composed of high minute uninjured D1 female soccer athletes was shown to negatively respond to competitive season stress at both MID and POST time points with >24 h rest and >48 h post-competition without affecting MVP (*p* = 0.75). The accumulation of training stress on neuromuscular function was demonstrated with the decline in NCOMP/NANT reach distance performance across 6 weeks of competition (PRE:MID), which then increased MID:POST. This study demonstrates the sensitivity of the neuromuscular system to accumulated training stress and thus injury predisposition with and without adequate rest (POST season and MID season, respectively). Interestingly, despite seven weeks of additive competition stress (MID:POST), extended rest (11-days rest before POST testing; Figure 1) restored NCOMP/NANT reach distance to PRE values. However, NANT and NCOMP segmental comparisons increased MID:POST despite rest demonstrating the plasticity of competitive season stress on neuromuscular function.

Maximal vertical power was unaffected across the competitive season indicating that the cumulative stress of the competitive season had no significant impact on the neuromuscular system's ability to produce force within our population with >24 h of rest (Figure 3). Previous research found that neuromuscular function is affected by training stress that impacts voluntary maximal power (Brownstein et al., 2017; Thomas et al., 2018). However, neuromuscular function was shown to fully recover within 72 h after a single soccer match (Brownstein et al., 2017) which our results disagree. It is noteworthy that Brownstein et al. (2017) examined the acute effects of a single 90 min competitive soccer match on central and peripheral indices where this study focused on the accumulation of training stress across the competitive season (with >48 h of rest post-competition). Previous research show that the accumulation of competitive stress can have negative effects on performance and can perpetuate injury (Bengtsson et al., 2013; Silva et al., 2016; Jones et al., 2017; Dubois et al., 2018; Walker et al., 2019). Our findings indicate that MVP can maintain/recover within 24 h despite the accumulation of training stress incurred throughout the competitive season (Jones et al., 2017; Dubois et al., 2018; Walker et al., 2019; Satkunskiene et al., 2020), which disagrees with the current literature (Silva et al., 2016; Brownstein et al., 2017; Thomas et al., 2018). Interestingly, CNS activity specific to the motor cortex has been shown adapt to fatigue which has previously been shown to occur acutely to maintain force production (Jiang et al., 2012). While significant changes in MVP were not observed in our population, movement quality decreased from PRE:MID and increased MID:POST (NCOMP/NANT reach distance) suggesting that chronic training stress and the negative indices of injury are perpetuated by mobility/stability disfunction without affecting MVP (Figure 3). Therefore, evaluating movement throughout the competitive season may be a more sensitive measure of chronic neuromuscular fatigue to assess injury risk, which can be a result of motor control degradation separate from the CNS. It is important to note that the degradation of motor control can manifest as a function of deteriorating PNS function: muscle tone, vestibular control, peripheral fatigue, etc. (Needle et al., 2014). However, our population is represented by uninjured athletes who maintained the ability to produce maximal force with diminished neuromuscular control that can facilitate greater injury predisposition among athletes (Brazier et al., 2019; Higashihara et al., 2019).

The YBT indices (NCOMP, NANT, LLSYM) observed across the competitive season to represent neuromuscular function have been shown to independently evaluate injury risk as they focus on different aspects of lower body neuromuscular integrity (Plisky et al., 2006; Gonell et al., 2015; Stiffler et al., 2017). Most apparent was the left and right NCOMP/NANT reach distance decreasing after 6 weeks of competition. More concerning was that 80% of the population (12/15 athletes) experienced a bilateral decline in NCOMP reach distance from PRE:MID dropping NCOMP reach distance below the risk threshold (<94% total reach distance) (Figure 4), which increased injury risk 6.5 × MID season (Plisky et al., 2006; Gonell et al., 2015; Stiffler et al., 2017) (Figure 4). Additionally, bilateral NANT reach distance scored below the injury risk threshold (NANT reach <84% of limb length) over the entire competitive season, which further reduced and paralleled the significant NCOMP reach reduction PRE:MID (*p* < 0.05). The NCOMP and NANT reach distance reductions PRE:MID reinforce the effects of accumulated competitive season stress on the neuromuscular function (Bengtsson et al., 2013; Brownstein et al., 2017; Dubois et al., 2018; Satkunskiene et al., 2020). Increased passive muscle tone (Masi and Hannon, 2008) that disrupts the agonist/antagonist relationship (Iwamoto et al., 2017; Higashihara et al., 2019) can corrupt postural control and therefore limit mobility; a necessary function in competition. Interestingly, NCOMP reach distance increased MID:POST which would suggest restored neuromuscular function and decreased injury risk (Gonell et al., 2015; Stiffler et al., 2017). However, L/R asymmetry increased >4.0 (Figure 3; Table 2) over the same period indicating increased segmental neuromuscular dysfunction and therefore heightened injury risk (Plisky et al., 2006; Gonell et al., 2015). Left-right reach asymmetries are postulated to occur as a result of ankle dorsiflexion limitations as well as deficits in strength, flexibility, and motor control that inform injury risk (Needle et al., 2014; Stiffler et al., 2017). A limitation of this study was the delayed collection of the POST time block data and associated rest/recovery extension that occurred 11 days post-final match to comply with NCAA guidelines (The National Collegiate Athletic Association, 2018). However, while POST-season NCOMP/NANT reach distances increased to PRE-season values with extended rest, L/R NCOMP reach asymmetry increased MID:POST as a result training stress accumulation despite adequate recovery. These observations illustrate the plastic nature of unilateral NCOMP asymmetry to accumulated training stress after onset which can affect acceleration, deceleration, and lateral explosive movements perpetuating injury. While segmental changes paralleled each other in response to accumulated training stress across time in this study, evaluating dominant/non-dominant segmental neuromuscular responses to training stress could help describe the segmental differences that inform injury risk observed in this study and past research (Stiffler et al., 2017).

Normalized anterior reach (NANT) is shown to be an indicator of lower extremity mobility/stability while revealing potential limitations that preclude normal function and promote greater injury predisposition (Plisky et al., 2006; Gonell et al., 2015; Stiffler et al., 2017). Left-right NANT reach distance comparisons from PRE:MID season significantly decreased which promoted greater injury predisposition as a consequence to initiation of competitive season training stress. Furthermore, NANT asymmetry (absolute difference) increased by 28% beyond the 4.0 injury risk threshold (Plisky et al., 2006; Gonell et al., 2015; Stiffler et al., 2017) from MID to POST (Figure 4; Table 2) further demonstrating the plasticity of side-to-side asymmetries despite 11 days of rest. Comparatively, NANT reach decreased and then increased PRE-MID-POST with recovery illustrating the elasticity of reach distance to recovery. The average NANT decrement across the season was 24.6% below the aforementioned injury risk threshold. Aggregate data show that 100% of the population exhibited a >2.5 × (Plisky et al., 2006; Gonell et al., 2015; Stiffler et al., 2017) elevated injury risk (NANT reach <84% limb length) (Plisky et al., 2006) for both limbs at all three time points (Figure 4) despite the increase in reach distance MID:POST. Practitioners should consider the sensitivity of adequate reach distance and side-to-side asymmetries to evaluate chronic neuromuscular fatigue. Therefore, the reach limitations observed in non-injured athletes can be an indicator of heightened passive muscle tone through amplified lower extremity neuromuscular agonist/antagonist activity restricting mobility (Needle et al., 2014; Iwamoto et al., 2017; Higashihara et al., 2019).

Greater co-contraction of ankle joint stabilizers are shown to limit range of motion in accelerated movement patterns in healthy subjects (Iwamoto et al., 2017). While the YBT utilizes slow, controlled movements to assess mobility and stability, previous research indicates that heightened muscular tonicity (abnormal increase in muscle activity) can be a limitation to mobility (Needle et al., 2014; Higashihara et al., 2019; Satkunskiene et al., 2020). The non-injured subjects in this study started the competitive season (PRE) with bilateral NANT reach limitations and a heightened injury predisposition that worsened PRE:MID and were restored MID:POST season. The results MID & POST occurred despite >24 h of rest prior to MID and a 11-day recovery prior to POST testing. While peripheral neural function including muscle electrical activity (EMG) was not measured in this study, non-injured players that have compromised lower extremity mechanical function while maintaining MVP is suggestive of neuromuscular tone dysfunction that can perpetuate injury (Needle et al., 2014; Higashihara et al., 2019; Satkunskiene et al., 2020). Acknowledging that strength is a necessary component of stability (Needle et al., 2014; Stiffler et al., 2017), our population maintained peak power suggesting strength was not a limitation. Nevertheless, specific aspects of the YBT, namely NCOMP/NANT reach distance and symmetry should be monitored regularly as chronic training clearly affects neuromuscular function, particularly during periods of high training stress to help mitigate injury risk (Bengtsson et al., 2013; Silva et al., 2016; Satkunskiene et al., 2020).

Additional metrics of the YBT included LLSYM that was previously identified as an injury risk indicator (Plisky et al., 2006; Gonell et al., 2015; Stiffler et al., 2017). This study evaluated the chronic nature of competitive season stress with >24 h of rest which found no significant LLSYM changes. NCOMP and NANT metrics rather than pelvic dysfunction may be more sensitive indicators of chronic stress in collegiate female soccer players. However, past research has demonstrated that LLSYM and therefore pelvic symmetry dysfunction does occur in collegiate athletes (Giles et al., 2017). Notably, the effects of chronic training stress has been shown to reside for weeks after training cessation (Nédélec et al., 2012; Silva et al., 2016) despite reductions in training volume and intensity. However, pelvic dysfunction (LLSYM) has been previously shown to diminish in collegiate female soccer players with extended rest (9 weeks of recovery) (Giles et al., 2017). The residual effects of extended high-volume high-intensity training inherent to the competitive season has lasting effects on neuromuscular function that are best mitigated through adequate rest. While athletes' rest is regulated throughout the competitive season, suitable transition period management as well as pre-season preparation (Silva et al., 2016) is necessary to mitigate non-contact soft tissue injury (Plisky et al., 2006; Gonell et al., 2015; Stiffler et al., 2017; Higashihara et al., 2019).

Postural degradation has been shown to occur in heathy populations due to neuromuscular dysfunction (Masi and Hannon, 2008; Needle et al., 2014; Stecco et al., 2014; Higashihara et al., 2019) that contribute to injury (Plisky et al., 2006; Gonell et al., 2015; Stiffler et al., 2017; Higashihara et al., 2019). Our population consisted of uninjured healthy athletes with no apparent joint or mechanical dysfunction preventing testing. Therefore, alterations in neuromuscular function that manifest as a result of accumulated competitive season stress may explain degradation in mobility and stability (Plisky et al., 2006; Masi and Hannon, 2008; Brazier et al., 2019; Higashihara et al., 2019) while absent of power deficiencies that are necessary to perform. It is important to note that multiple systems contribute to stability, some of which were not tested. However apparent this limitation is, the fact remains that accumulated training stress (Jones et al., 2017; Dubois et al., 2018; Walker et al., 2019) has been shown to effect stability and mobility across the competitive season which should be further investigated with more stringent laboratory methodologies. Additional considerations are that bilateral movements such as running rely on reciprocal contributions of both limbs and are suggestive of normal function and therefore maintenance of performance. However, unilateral stability/mobility testing can expose masked neuromuscular dysfunction that can lead to limitations in joint stability and thus range of motion (Plisky et al., 2006) that promote injury risk. Therefore, the sensitivity of the YBT may be more relevant to evaluate changes in neuromuscular function rather than performance measures. Furthermore, the applied nature and objectivity along with the low cost and portability make the YBT a relevant objective field test for practitioners.

## Conclusion

The accumulation of competitive season training stress was shown to affect neuromuscular function after 6 weeks and persist despite rest by impacting unilateral lower extremity stability. The NCOMP and NANT limitations observed are indictive of altered neuromuscular stability function and are more sensitive to the effects of accumulated training stress rather than power-based measures. Normalized composite scores measure anterior, posteriorlateral, and posteriormedial reach and are indicators of mobility/stability while anterior reach considers ankle dorsiflexion. The YBT through single limb loading stresses unilateral neuromechanical coordinated movement of the lower extremity exposing motor control deficiencies that limit range of motion. Throughout the competitive season, increased passive muscle tone may be the cause of the observed decrement in NANT/NCOMP reach paired with asymmetries within our population. Reach distance was shown to decrease and recover after rest. However, asymmetries persisted despite rest demonstrating the plasticity of accumulative competition training stress on neuromuscular function with recovery predisposing healthy, uninjured athletes to 6.5-fold greater injury risk. Furthermore, the degradation in postural control while maintaining maximal power can in turn lead to greater injury predisposition throughout the competitive season. These findings suggest that training stress over time absent adequate rest can negatively impact neuromuscular function and increase lower extremity injury risk due to less compliant tissues (elevated passive muscle tone). In summary, left and right NCOMP/NANT asymmetries and reach distances should be considered and monitored regularly when evaluating the impact of competitive season training stress as a means to reduce injury risk.

## Data Availability Statement

The raw data supporting the conclusions of this article will be made available by the authors, without undue reservation.

## Ethics Statement

The studies involving human participants were reviewed and approved by Longwood University Institutional Review Board: HHS IRB number: IRB00008677 FWA number: FWA00019433. The patients/participants provided their written informed consent to participate in this study.

## Author Contributions

TP and KL: Conceptualization and formal analysis. TP: Methodology and project administration. TP, JG, and KL: Writing. TP, KL, JG, CM, and LB: Data collection. All authors have read and agreed to the published version of the manuscript.

## Conflict of Interest

The authors declare that the research was conducted in the absence of any commercial or financial relationships that could be construed as a potential conflict of interest.
